# A Functional Role for 4qA/B in the Structural Rearrangement of the 4q35 Region and in the Regulation of *FRG1* and *ANT1* in Facioscapulohumeral Dystrophy

**DOI:** 10.1371/journal.pone.0003389

**Published:** 2008-10-13

**Authors:** Iryna Pirozhkova, Andrei Petrov, Petr Dmitriev, Dalila Laoudj, Marc Lipinski, Yegor Vassetzky

**Affiliations:** 1 Université Paris-Sud 11, CNRS UMR 8126, Interactions moléculaires et cancer, Institut de Cancérologie Gustave-Roussy, Villejuif, France; 2 INSERM, ERI25, F-34000, Montpellier, France, Université Montpellier 1, Montpellier, France; The Babraham Institute, United Kingdom

## Abstract

The number of D4Z4 repeats in the subtelomeric region of chromosome 4q is strongly reduced in patients with Facio-Scapulo-Humeral Dystrophy (FSHD). We performed chromosome conformation capture (3C) analysis to document the interactions taking place among different 4q35 markers. We found that the reduced number of D4Z4 repeats in FSHD myoblasts was associated with a global alteration of the three-dimensional structure of the 4q35 region. Indeed, differently from normal myoblasts, the 4qA/B marker interacted directly with the promoters of the *FRG1* and *ANT1* genes in FSHD cells. Along with the presence of a newly identified transcriptional enhancer within the 4qA allele, our demonstration of an interaction occurring between chromosomal segments located megabases away on the same chromosome 4q allows to revisit the possible mechanisms leading to FSHD.

## Introduction

Facio-scapulo-humeral muscular dystrophy (FSHD) is an autosomal dominant neuromuscular disease characterized by weakness and atrophy of muscles of the face, upper arms and shoulder girdle. In patients with FSHD, a deletion in a polymorphic locus of chromosome 4q reduces the number of D4Z4 repeats to less than 10 vs up to 200 in normal individuals [1]. Each 3.3 kbp D4Z4 element harbors *DUX4*, a gene which encodes a double homeodomain protein [2]–[4]. Three other genes *FRG1* (FSHD Region Gene 1) [5], [6], *FRG2* (FSHD Region Gene 2) [5], [7] and *ANT1* (Adenine Nucleotide Translocator 1) [8] are located within the 4q35 chromosomal region and have been reported to be upregulated in FSHD patients. Aberrant expression of *FRG1*, which is thought to encode a splicing regulator [6], [9], could explain the simultaneous changes in expression of many genes. Nevertheless, the evidence of their involvement in FSHD pathogenesis is missing. Some studies even argue against the upregulation of *FRG1* and *FRG2* in FSHD muscles [10], [11]. Indeed, to date, the many proteomics and transcriptome approaches have provided a wealth of data suggesting that the contraction of the D4Z4 repeat array is not sufficient to cause the disease and that FSHD is likely to be a multifactorial disorder (reviewed in [12]).

Several years ago a transcriptional repressor was identified within the D4Z4 repeat array [5]. However, we have recently demonstrated that overall, each D4Z4 repeat has an enhancer activity due to the presence of a very strong enhancer [13]. Moreover, we have shown that a nuclear matrix attachment site (S/MAR), which is positioned in the immediate vicinity of the D4Z4 repeat array [14], may function as an insulator and block the D4Z4 enhancer in normal, but not FSHD, cells [13]. In fact, this S/MAR is prominent in normal myoblasts and non-muscular human cells, and much weaker in muscle cells derived from FSHD patients [14]. From this observation, we inferred that, in normal human myoblasts, the D4Z4 repeat array and neighboring genes are located in two distinct loops, whereas, in myoblasts from FSHD patients, they are in a single one. This suggests that a looping mechanism could lead to a direct contact between the D4Z4 array and genes that are positioned in *cis* on the chromosome but are too far away to be subjected to transcriptional regulation through classical molecular mechanisms [14].

Intriguingly, FSHD occurs only in individuals bearing the 4qA allele. 4qA/B is a 10 kb-long polymorphic segment directly adjacent to the D4Z4 repeat array. It exists in two allelic forms, 4qA and 4qB, which are 92% identical and equally common in the general population [15], [16]. The main difference between the two alleles resides in a tract of β-satellite repeats present in 4qA but not 4qB [15]. This dissimilarity may bear consequences either in the predisposition to deletions occurring within the D4Z4 repeat array or in the structural consequences of the deletion.

Here, we have further investigated the three-dimensional structure of the 4q subtelomeric region using the recently described 3C technique. We now report significant differences existing between FSHD and normal muscle cells.

## Results

### 3C analysis of DNA-DNA interactions at 4q35 in normal human myoblasts

The 3C technique evaluates the spatial proximity of two genomic fragments based upon their relative propensity to get crosslinked *in vivo* [17]–[19]. The method uses a restriction enzyme to digest previously crosslinked chromatin. After ligation of very dilute DNA to favor intramolecular rather than intermolecular ligation of crosslinked DNA, the ligated fragments are amplified by PCR using specifically designed primers. In the present study, we have used the *Bgl*II enzyme whose recognition sequence is present on average every 3,500±1,500 bp within the studied region. Such a DNA length is appropriate for the 3C assay.

**Figure 1:**
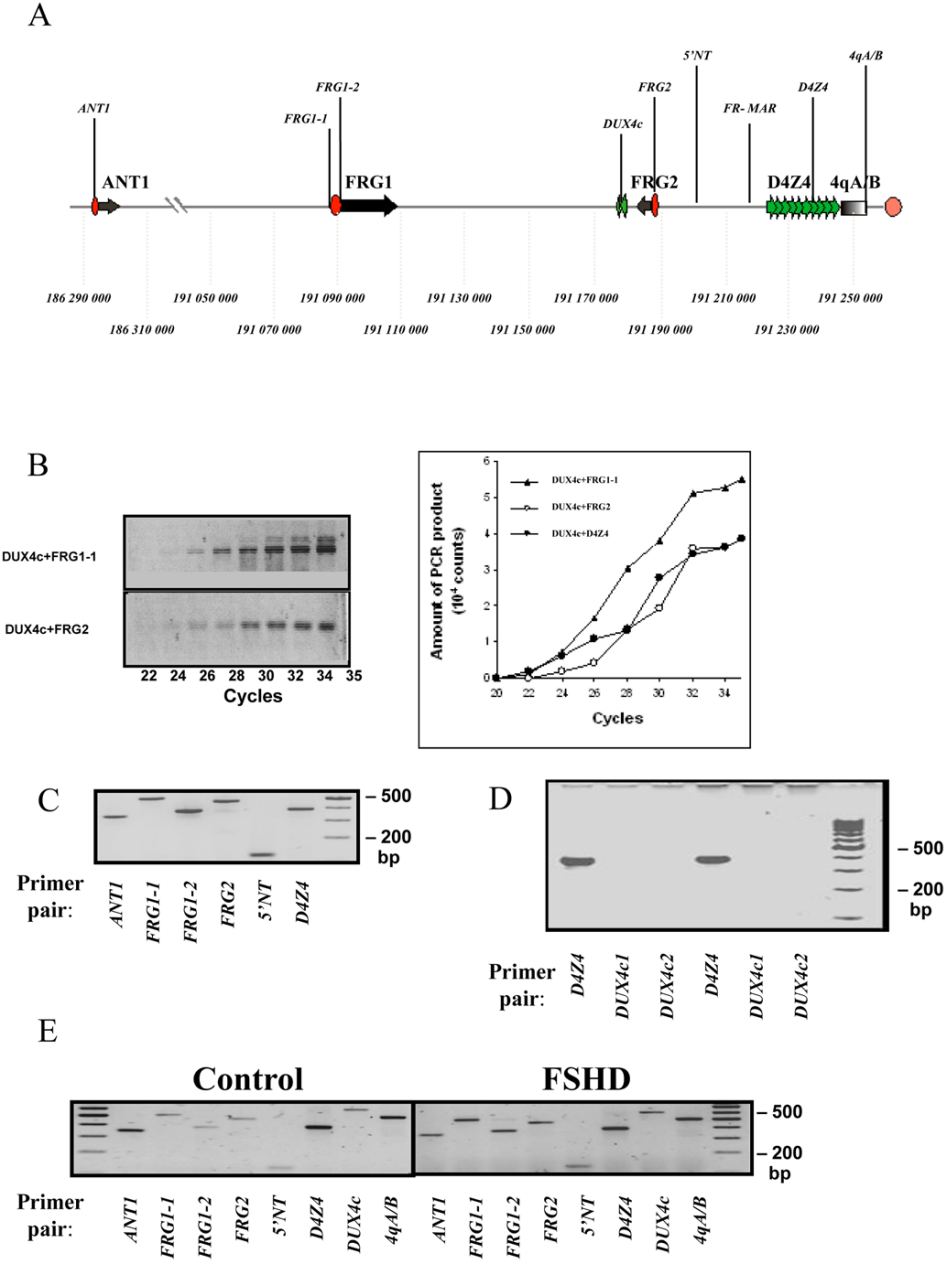
3C analysis of nine landmarks in the 4q35 region. A. Map and genomic coordinates (in bp) of primer pairs used for the 3C analysis. Genes are represented by unique arrows, promoters by ovals. The D4Z4 array is shown as green block arrows. B. Control digestion on crosslinked templates. Genomic DNA was digested with *Bgl*II and amplified using the primer pairs that allow only the amplification of non-digested DNA. No PCR products were observed in the absence of the ligation step. C. The PCR amplification linear range was obtained by titration of the template concentration and number of amplification cycles. Finally, 10 ng of crosslinked template and 100 ng of control template in 15 µl of reaction mixture were used in our experiments. The PCR cycling conditions were as follows: 94°C for 3 min; 94°C for 45 sec and 58°C for 30 sec, 72°C for 50 sec, followed by a final extension at 72°C for 10 min using Taq DNA Polymerase (Invitrogen). D. The DNA GM10115A human/rodent hybrid cell line containing a single chromosome 4 was digested with *Bgl*II, ligated and then amplified using specific primer pairs to verify the accuracy of the primer pairs for the chromosome 4 sequences. E. The D4Z4 repeat cloned into the pGEM42 plasmid was amplified using one primer pair specific for D4Z4 and two different primer pairs specific for DUX4c (DUX4c1 and DUX4c2). Two different template concentrations, 100 ng and 200 ng were used for amplification.

We selected several genes and landmarks (Figure 1A) within the 5 Mb-long subtelomeric region of chromosome 4q to study their propensity to get crosslinked *in vivo*. These included 4qA/B, a distal segment adjacent to the polymorphic 4qA/4qB marker [15], [16]; D4Z4, a 3.3 kb fragment containing the D4Z4 repeat array itself; FR-MAR, the fragment containing the S/MAR whose function is weakened in FSHD muscle cells [14]; 5'NT, a non-transcribed fragment located between the D4Z4 array and the *FRG2* gene; FRG2, the promoter region of *FRG2* [7]; DUX4c, a DNA fragment in the vicinity of the unique D4Z4 copy located between the *FRG2* and *FRG1* genes [4]; two fragments, FRG1-1 and FRG1-2, that correspond to the distal and proximal part of the *FRG1* gene promoter, respectively [20]; and ANT1, the promoter region of the *ANT1* gene [21](Figure 1A). We then designed specific PCR primers for each *Bgl*II restriction fragment as detailed in Materials and Methods.

We carried out preliminary experiments (Figure 1B–E) to define the optimal conditions for the 3C analysis. For PCR amplification we chose a number of cycles that fell into the linear range of amplification (Figure 1B, right panel). However, the 4q35 locus contains repetitive sequences and copies of the *FRG1* and *FRG2* genes also exist elsewhere in the genome [2]. We thus had to verify that the primer pairs used in this study specifically amplified genomic DNA from chromosome 4. To this aim we used genomic DNA extracted from the GM1015 human/rodent hybrid cell line in which chromosome 4 is the only human chromosome. Indeed, all six amplification products obtained using DNA from this cell line migrated identically to the control PCR products obtained from total human DNA (Figure 1C). We then verified the specificity of the primer pairs for DUX4c, a fragment with considerable homology to D4Z4 using the pGEM42 construct which contains two D4Z4 repeats and 5′ and 3′ flanking sequences, but no DUX4c sequence [22]. With this template we obtained an amplification product with the D4Z4 but not with the DUX4c specific primers (Figure 1D). Finally, we confirmed the sequence specificity of the DUX4c and DUX4 products by sequencing (data not shown), and verified that all primer pairs used produced specific fragments from total DNA of normal and FSHD myoblasts (Figure 1E).

**Figure 2:**
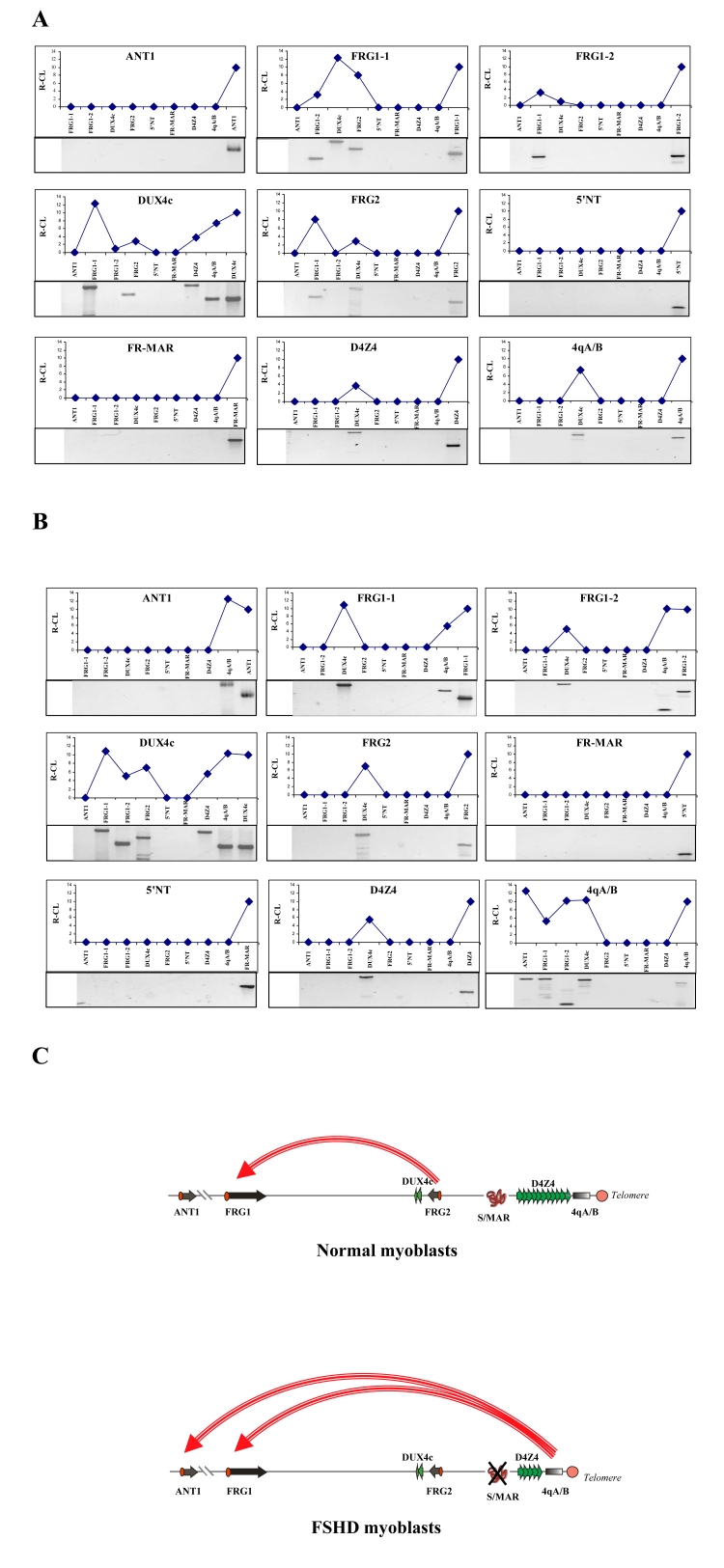
3C Analysis of the 4q35 locus. A–B. Representation of the spatial proximity in normal (A) and FSHD (B) myoblasts. The fragment tested for crosslinking is indicated in each panel. An arbitrary score of 10 corresponds to the PCR amplification obtained using primers located on either side of the restriction site separating two adjacent fragments within the corresponding genomic segment. The Y axis indicates relative levels of interaction with the other landmarks tested which are represented along the X axis according to their localization along chromosome 4q. The data represent the average results of three independent experiments. The panels below the charts show the 3C ligation products detected by PCR amplification using specific primers. One experiment out of three independent ones is represented in the Figure. C. The differences in 3C interactions between the normal (top) and FSHD myoblasts. Only interactions which are different between the normal and FSHD myoblasts are shown.

We next used the 3C assay to evaluate the spatial proximity of the selected 4q35 landmarks in normal human myoblasts (Figure 2A). We did not detect any interaction between *ANT1* and the other landmarks (Figure 2, upper left panel). This indicates a lack of proximity between the *ANT1* gene and all other landmarks tested. This result was confirmed when the other landmarks were tested for proximity with *ANT1* (see the *ANT1* point, first on the left on the x-axis in all the other panels of Figure 2A). In contrast, we consistently detected an interaction between FRG1-1 and FRG2 and DUX4c. Specifically, DUX4c strongly interacted with the distal part of the promoter of *FRG1* (FRG1-1) and, to a lower extent, with the promoter of *FRG2*, and also with the subtelomeric region proximal to the 4qA/4qB marker. FR-MAR and 5'NT did not interact with other landmarks, whereas D4Z4 interacted only with the region proximal to DUX4c. Thus, in normal myoblasts, we have found that the D4Z4 repeat array does not directly interact with any gene promoter.

### 3C analysis of DNA-DNA interactions within 4q35 in FSHD myoblasts

We next performed the same 3C analysis using myoblasts derived from an FSHD patient. Differently from what observed in normal muscle cells, we could not detect any interaction between FRG1-1 and FRG2 or FGR1-2, whereas we consistently identified a novel interaction between FRG1-1 and 4qA/4qB (Figure 2B). Indeed, in FSHD myoblasts, the 4qA/B landmark strongly interacted not only with DUX4c (as in control cells), but also with FRG1-1, FRG1-2 and the promoter of the *ANT1* gene. This indicates that despite being located 5 Mb proximally on the 4q chromosome, the *ANT1* gene directly interacts with 4qA/B in the nuclear space of FSHD cells. This interaction was indeed specific as ANT1 did not crosslink with any other sequence but 4qA/B. Additional differences also exist between normal and FSHD cells regarding 4qA/B whose interactions with FRG1-1 and FRG1-2 were also FSHD-specific. As in control cells, we did not observe any interaction between FR-MAR or 5′NT and the other landmarks, whereas the D4Z4 repeat directly interacted only with DUX4c, but not with any of the gene promoters.

The major differences in the 3D organization of the 4q35 locus between normal and FSHD myoblasts are summarized in Table 1 and Figure 2C.

**Table 1:** Frequencies of cis-interactions within 4q35 in normal and FSHD myoblasts.

		*ANT1*	*FRG1-1*	*FRG1-2*	*DUX4c*	*FRG2*	*5*'*NT*	*FR-MAR*	*D4Z4*	*4qa/b*
	Primer	1	2	3	4	5	6	7	8	9
***ANT1***	**1**	+								|
***FRG1-1***	**2**		+	−	+	−				|
***FRG1-2***	**3**		−	+	+					|
***DUX4c***	**4**		+	+	+	+			+	+
***FRG2***	**5**		−		+	+				
***5*** **'** ***NT***	**6**						+			
***FR-MAR***	**7**							+		
***D4Z4***	**8**				+				+	
***4qA/B***	**9**	|	|	|	+					+

Horizontal and vertical dashes indicate interactions detected by the 3C technique in normal and FSHD myoblasts, respectively.

− Normal Myoblasts.

| FSH Myoblasts.

### The majority of the interactions detected in the 3C assay occur in *cis* within 4q35

**Figure 3:**
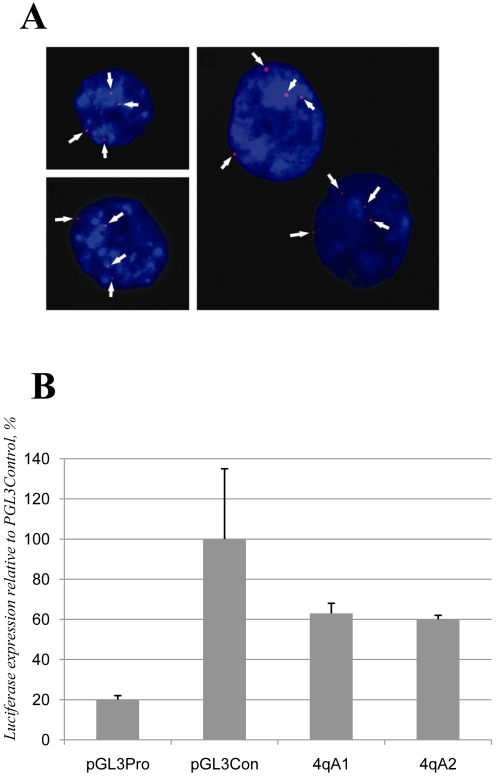
A. FISH analysis on primary human myoblasts. Nuclei from normal (left panels) and FSHD (right panel) primary myoblasts were hybridized to a FR-MAR probe (arrowed dots) and counterstained with DAPI. B. The 4qA allele contains a transcriptional enhancer. The transcriptional effect of the 4qA allele was tested 48 hrs after transfection in HeLa cells. The enhancer strength was quantified relative to the luciferase activity generated by the pGL3 plasmid with the SV40 enhancer (pGL3Con). Equal amounts of the plasmids were transfected. Luciferase signals were normalized to the total protein content in the extracts. pGL3Pro, enhancer-less, empty pGL3 plasmid; 4qA1 and 4qA2, 4qA allele cloned in the enhancer-less pGL3 plasmid.

The data obtained with the 3C assay evidence the spatial proximity of sequences along the subtelomeric region of chromosome 4q. However, approximately 60 kbp of sequences within this region are also present on chromosome 10q which contains a region homologous to a 4q35 segment [23]. Thus, the interactions detected by the 3C assays could have occurred in *trans* between chromosomes 4q and 10q rather than in *cis* within 4q. To investigate this possibility, we measured the proximity of the homologous 4q and 10q regions. To this aim, we used the FISH technology to localize the long arms of chromosome 4 and 10 in interphase nuclei (Figure 3A). Some hybridization signals were in direct contact with each other. In this case, we assumed that somatic pairing did take place. From the analysis of 200 nuclei, the level of somatic pairing ranged between 9 and 10.5% of all signals in both control and FSHD myoblasts (Table S2). This was consistent with the low level of pairing (4.5%) reported between chromosomes 4 and 10 in a previous study [24]. The higher pairing level observed here corresponds to the fact that, in addition to the 4q–10q interactions, we have also revealed contacts between homologous chromosomes (4q-4q and 10q-10q). From these results we can conclude that, although the existence of interactions in *trans* cannot be completely excluded, these do not occur in more than the 10% of the nuclei, whereas in 90% of the nuclei, the loci of interest are too far away from each other to interact. Therefore, the interactions detected by our 3C experiments mainly reflect interactions occurring in *cis* within 4q35.

### The 4qA allele contains a transcriptional enhancer

We then asked whether the 4qA/B marker, which in FSHD myoblasts interacts directly with the promoters of *FRG1* and *ANT1*, could have a role in the transcriptional regulation of these two genes. Since all FSHD patients carry the 4qA phenotype on the deleted 4q chromosome [15], we tested whether the 4qA allele could directly regulate gene transcription. To this aim we cloned the 4qA marker in both orientations in the pGL3-promoter plasmid, a luciferase reporter vector. We transfected constructs and control plasmids in HeLa cells, and then measured reporter gene expression 48 hours after transfection. The presence of the SV40 enhancer in the positive control (pGL3con, Figure 3B) resulted in a five-fold increase of the transcription levels in comparison to the enhancer-less control plasmid (pGL3Pro). The 4qA fragment cloned into the enhancer-less pGL3–promoter plasmid stimulated luciferase synthesis with 60% efficiency as compared to the SV40 enhancer positive control. Thus, the 4qA allele exhibited properties of a transcriptional enhancer. This enhancer was also active in a cell line derived from a human rhabdomyosarcoma, a tumor of muscular origin (data not shown).

## Discussion

Despite many studies performed in the last twenty years, the mechanism leading to the emergence of FSHD remains poorly understood. The 3C data reported here provide the first experimental evidence that, in this genetic disease, molecular events occur that involve chromosomal segments located at a very large linear distance on the partially deleted chromosome 4q. Specifically, we have observed that in FSHD myoblasts, the subtelomeric 4qA/B marker strongly interacts with the promoter of the *FRG1* gene which is located dozens of kbp proximally on the chromosome, depending on the number of remaining D4Z4 repeats. Even more strikingly, we documented a direct interaction of 4qA/B with the promoter of the *ANT1* gene which lies at a linear distance greater than 5 Mbp on the centromeric side. This interaction is FSHD-specific as, in control myoblast cells, the 4qA/B marker did not interact with the *FRG1*, or the *ANT1* promoters.

4qA/B is a 10 kb-long polymorphic segment directly adjacent to the D4Z4 repeat array. It exists in two allelic forms, 4qA and 4qB, which are 92% identical and equally common in the general population. FSHD, however, has been reported to occur only in individuals with the 4qA allele [15], [16]. The main difference between the two alleles resides in a tract of β-satellite repeats present in 4qA but not 4qB [15]. This difference may bear consequences either in the predisposition to deletions occurring within the D4Z4 repeat array or in the pathological consequences thereof.

Another surprising observation was that, in both normal and FSHD cells, the D4Z4 marker interacted only with its related sequence DUX4c among the various segments tested. No interactions were detected with the promoter regions of *ANT1*, *FRG1* or *FRG2*. In accordance, the hypothesis of a transcriptional regulation through a direct contact of the D4Z4 array with the promoters of these three genes [5], [7], [13], [14] appears unlikely. DUX4 and DUX4c are two genes that have been shown to be transcribed within the D4Z4 repeats [2], [3], [25]. Thus, our results suggest that the D4Z4 enhancer, within the D4Z4 repeat array, may directly regulate the transcription of the *DUX4* and *DUX4c* genes.

We then found that DUX4c crosslinked with the FRG1 and FRG2 promoter regions in both normal and FSHD myoblasts (Figure 2). We therefore postulate that DUX4c plays a key role in the three-dimensional organization of the locus. Sequence alignment analysis (data not shown) suggests that DUX4c contains a transcriptional enhancer. Moreover, DUX4 interacts with DUX4c which, in turn, makes contact with *FRG1* and *FRG2*. This may provide a molecular basis for the transcriptional regulation of neighbor genes by DUX4/DUX4c.

We then detected a new enhancer element in the 4qA allele that may regulate the expression of the *FRG1* and *ANT1* genes specifically in FSHD cells through a direct interaction with the respective gene promoters. Indeed, both *ANT1* and *FRG1* are activated in FSHD patients [5], [6], [8]. It is noteworthy that the (1.5 to 3 fold) up-regulation of these two genes seen in FSHD patients is consistent with the relatively weak effect of the 4qA enhancer in the luciferase assay.

Recently, we have reported that in FSHD myoblasts, the nuclear matrix attachment site FR-MAR was specifically delocalized from the nuclear matrix [14]. In normal cells, this S/MAR may constrain the flexibility of the region by anchoring it to the nuclear matrix, thus restricting interactions of adjacent sequences in the three dimensional nuclear space. This could particularly affect the 4qA/B marker which is separated from neighbor genes by the S/MAR. In FSHD cells, the delocalization of FR-MAR would thus result in an increased flexibility of the corresponding chromosomal segment and additional possibilities of interaction for the 4qA/B marker. This may provide an explanation for the FSHD-specific, direct interaction of 4qA/B with the *ANT1* and *FRG1* gene promoters we observed in FSHD myoblasts. In the present 3C experiments, no interactions were detected that involved FR-MAR. This should not be surprising since previous 3C studies have already stressed that S/MARs appear to interact only with other SMARs [26].

**Figure 4:**
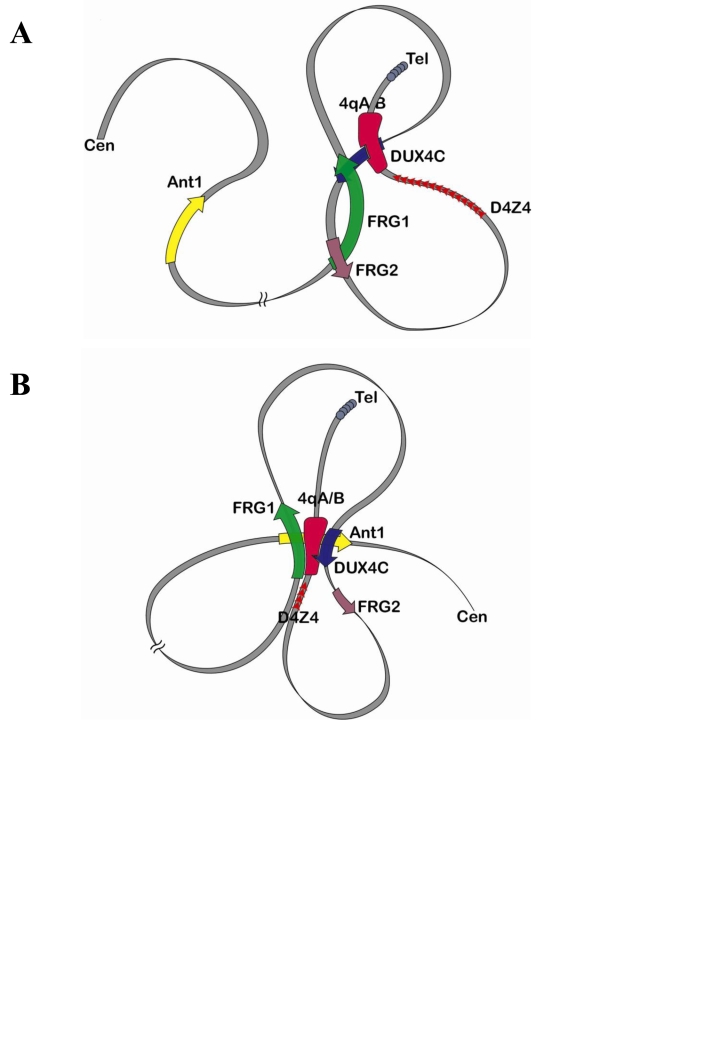
A–B. Models schematizing the proximity between 4q subtelomeric fragments in control (A) and FSHD (B) nuclei.

The experimental approach used here provides new ways to systematically explore the higher-order chromatin structure of any chromosomal region. In this study, we have found that the binding of DUX4c to the *FRG1* and *FRG2* gene promoters appears to play a key role in structuring the 4q35 region in normal cells. Other interactions take place in FSHD cells and this is the likely result of a global reorganization of the locus in relation with the contraction of the number of D4Z4 tandem repeats. This reorganization is schematized in the three dimensional model shown in Figure 4. In FSHD cells (Figure 4B), the deletion of D4Z4 repeats and the delocalization of the proximal S/MAR would result in the formation of a giant loop where the subtelomeric 4qA/B sequence is now brought in close proximity not only to *DUX4C* and *FRG1* but also to the proximal *ANT1* gene promoter which lies 5 Mbp away on the centromeric side of the region. This major structural rearrangement, as compared to the normal situation (Figure 4A), would make gene promoters accessible to the DUX4c and 4qA enhancers specifically in FSHD myoblasts. One hypothesis to explain how such long-range changes in higher order chromatin structure can occur relates to differences in the methylation status of the corresponding regions [27], [28]. Further studies are clearly needed to explore this and other hypotheses.

## Materials and Methods

### Cell lines

The HeLa cell line was purchased from the ATCC collection. The GM10115A hybrid murine cell line containing the human chromosome 4 was a kind gift of Dr. Rosella Tupler. Primary muscle fibroblasts from two different healthy individuals and two FSHD patients with 5.5 D4Z4 repeats and 7 repeats [14], [29] in the 4q35 array, respectively, were cultured on a collagen-coated support in DMEM supplemented with 20% bovine fetal serum.

### 3C assay

The 3C assay was performed as described elsewhere [30] with some specific adaptations for myoblast cells. Nuclei were prepared using 2 volumes of ice-cold MES lysis buffer [31] for 1 volume of packed cells; a protease inhibitors cocktail (Roche, Complete Mini) was added immediately prior to use. The lysis of nuclei was checked under a microscope. Formaldehyde (Sigma) was added to diluted nuclei (final concentration of 1×10^−7^ /ml) to perform the crosslinking. Nuclei were then diluted tenfold and digested overnight at 37°C with *Bgl*II (New England BioLabs). The *Bgl*II restriction sites occur with an average frequency 3500 bp±1500 bp within the 4q35 locus, which is appropriate for the 3C assay.

Digestion mix was inactivated by adding SDS and digested DNA ligated overnight at a low concentration with T4 DNA ligase (Fermentas). Ligation products were detected by PCR amplification using fragment-specific primers. PCR products were separated on 2% agarose gels; images acquired using a Bio-DOC apparatus (Vilbour-Lourmat, France) and quantified using the Image Gauge 4.0 software (Fuji, Japan).

Three independent controls were carried out using genomic DNA from FSHD myoblasts, normal myoblasts and from the murine hybrid cell line containing the human chromosome 4 as the only human material. The DNA fragments spanning the *Bgl*II restriction sites were mixed in equimolar amounts as described elsewhere [18] and added to the appropriate non-crosslinked genomic DNA.

Relative crosslinking frequencies for combinatorial interactions were calculated as the ratio of the amount of product detected with crosslinked DNA template to the amount of product obtained with non-crosslinked, control DNA templates [17], [30]. The experiments were carried out in triplicate and were averaged. Data from two independent experiments are presented.

### 3C primer design

The primers spanning the *Bgl*II sites were designed using OLIGO Primer Analysis Software 6.71 at positions shown in Figure 1A. Primer sequences are shown in Table S1.

### FISH analysis

The p13E11 probe was derived from the pGEM42 plasmid [22] and labeled with biotin-14-dCTP. Hybridization on slides was performed as described earlier [32] using anti-biotin mouse antibodies conjugated with AlexaFluor 488 (Invitrogen, USA). Nuclei were counterstained with 0,5 µg/ml 4,6-diamindo-2-phenylindole (DAPI) and mounted using Vectashield antifade mounting medium (Vector Laboratories, USA). Slides were examined under an Olimpus Provis fluorescence microscope with a 100× oil immersion objective and the appropriate filters. Images were captured with a CCD camera (Photometrics, USA), using the RSImage software (Scanalytics, USA).

### Vectors and cloning

A series of pGL3 vectors (Promega, USA) was used for transient transfection studies. The pGL3-Promoter vector contains an SV40 promoter upstream of the luciferase gene. The pGEM42 plasmid containing the fragment of chromosome 4 corresponding to the allelic variant 4qA [22] (a kind gift of Dr. A.Belayew) was digested by BamHI and EcoRI (Fermentas, Lithuania). The 598 bp fragment was blunt-ended by Klenow (Fermentas, Lithuania) and cloned in two orientations upstream of the promoter region of the reporter plasmid pGL3-Pro (Promega, USA) digested by SmaI resulting in the plasmids pGL3-4qA1 and pGL3-4qA2.

The pGL3-Control vector contains the SV40 promoter and enhancer sequences, resulting in strong expression of the reporter gene in many types of mammalian cells. Therefore, it was used as a positive control in the experiments on the identification of a putative enhancer within the D4Z4.

### Luciferase assay

HeLa cells were plated in 24 well/plates 24 hours before transfection at the density of 50.000 cells per well. The plasmids used for transfection were purified with the Nucleobond midiprep kit (Macherey Nagel, Gremany) and 1 µg of each was transfected using JetPEI (Polyplus Transfections Inc., USA). 48 hours after transfection luciferase activity was measured with the Luciferase Assay System (Promega, USA) using a Microlourmat LB96P luminometer. The protein content of cell extracts was determined with the QuantiPro BCA assay kit (Sigma, USA). Each transfection was repeated at least 3 times.

## Supporting Information

Table S1 Primers used for the 3C assay.
(0.04 MB DOC)

Table S2 Frequency of pairing between the 4q and 10q in nuclei of normal and FSHD myoblasts.
(0.03 MB DOC)
