# Supplementary material for: A Functional Role for 4qA/B in the Structural Rearrangement of the 4q35 Region and in the Regulation of FRG1 and ANT1 in Facioscapulohumeral Dystrophy
Source: PLoS One. 2008 Oct 13;3(10):e3389. doi: 10.1371/journal.pone.0003389 (PMC2561064; doi:10.1371/journal.pone.0003389)
Supplement: Table S1 — Primers used for the 3C assay. (0.04 MB DOC) [file pone.0003389.s001.doc]

**Table S1.** Primers used for the 3C assay.

| Primers 1 | **ANT1** |
| --- | --- |
| forward | ACCCAAGCATGATATGG |
| reverse | TTGACTACTGCTGGAGTG |
|  |  |
| Primers 2 | **FRG1-1** |
| forward | ATGACTCCGGGCATCAT |
| reverse | TGCAGCGCTGGTGTGA |
|  |  |
| Primers 3 | **FRG1-2** |
| forward | GCTTGATATTGTTGGTGAGT |
| reverse | GACAACCGACTTCTACAAT |
|  |  |
| Primers 4.1 | **DUX4c-1** |
| forward | TGGCCCTTCGATTCTGA |
| reverse | CATGCATGTTCATAACGC |
|  |  |
| Primers 4.2 | **DUX4c-2** |
| forward | TGGCCCTTCGATTCTGA |
| reverse | GTGGAGGTGGTAGGTCTTT |
|  |  |
| Primers 5 | **FRG2** |
| forward | GTTGTTGTTGAGCCTGG |
| reverse | CCTAGAAGGTCACCGAA |
|  |  |
| Primers 6 | **NT** |
| forward | GGCTTGCACATAGGGT |
| reverse | CCTGCCTACAGATTAGC |
|  |  |
| Primers 7 | **FR-MAR** |
| forward | TTTCTGCTCCATTGTTCG |
| reverse | TAACTTGGAAACACAGCG |
|  |  |
| Primers 8 | **D4Z4** |
| forward | ACGGAGACTCGTTTGGA |
| reverse | TGGCCCTTCGATTCTGA |
|  |  |
| Primers 9 | **4qA/B** |
| forward | GCTGGAGTTACTTGGCT |
| reverse | AAGCCCATGAGAAAGAT |
