# Supplementary material for: A Functional Role for 4qA/B in the Structural Rearrangement of the 4q35 Region and in the Regulation of FRG1 and ANT1 in Facioscapulohumeral Dystrophy
Source: PLoS One. 2008 Oct 13;3(10):e3389. doi: 10.1371/journal.pone.0003389 (PMC2561064; doi:10.1371/journal.pone.0003389)
Supplement: Table S2 — Frequency of pairing between the 4q and 10q in nuclei of normal and FSHD myoblasts. (0.03 MB DOC) [file pone.0003389.s002.doc]

Table S2. Frequency of pairing between the 4q and 10q in nuclei of normal and FSHD myoblasts.

|  | FSHD | Control |
| --- | --- | --- |
| Number of nuclei counted | 200 | 200 |
| Numbers of nuclei with pairing observed | 21 | 18 |
| Pairing frequency, % | 10,5 | 9 |
